# Photodynamic Therapy: Targeting Cancer Biomarkers for the Treatment of Cancers

**DOI:** 10.3390/cancers13122992

**Published:** 2021-06-15

**Authors:** Xinning Wang, Dong Luo, James P. Basilion

**Affiliations:** 1 Department of Biomedical Engineering, Case Western Reserve University, 11100 Euclid Ave, Wearn Building B-49, Cleveland, OH 44106, USA; 2 Department of Radiology, Case Western Reserve University, 11100 Euclid Ave, Wearn Building B-44, Cleveland, OH 44106, USA; dxl576@case.edu

**Keywords:** photodynamic therapy, photosensitizer, targeted therapy, antibodies, ligands

## Abstract

**Simple Summary:**

Photodynamic therapy (PDT) is a minimally invasive treatment option that can kill cancerous cells by subjecting them to light irradiation at a specific wavelength. The main problem related to most photosensitizers is the lack of tumor selectivity, which leads to undesired uptake in normal tissues resulting in side effects. Passive targeting and active targeting are the two strategies to improve uptake in tumor tissues. This review focused on active targeting and summarizes recent active targeting approaches in which highly potent photosensitizers are rendered tumor-specific by means of an appended targeting moiety that interacts with a protein unique to, or at least significantly more abundant on, tumor cell surfaces compared to normal cells.

**Abstract:**

Photodynamic therapy (PDT) is a well-documented therapy that has emerged as an effective treatment modality of cancers. PDT utilizes harmless light to activate non- or minimally toxic photosensitizers to generate cytotoxic species for malignant cell eradication. Compared with conventional chemotherapy and radiotherapy, PDT is appealing by virtue of the minimal invasiveness, its safety, as well as its selectivity, and the fact that it can induce an immune response. Although local illumination of the cancer lesions renders intrinsic selectivity of PDT, most photosensitizers used in PDT do not display significant tumor tissue selectivity. There is a need for targeted delivery of photosensitizers. The molecular identification of cancer antigens has opened new possibilities for the development of effective targeted therapy for cancer patients. This review provides a brief overview of recent achievements of targeted delivery of photosensitizers to cancer cells by targeting well-established cancer biomarkers. Overall, targeted PDT offers enhanced intracellular accumulation of the photosensitizer, leading to improved PDT efficacy and reduced toxicity to normal tissues.

## 1. Introduction

**Figure 1:**
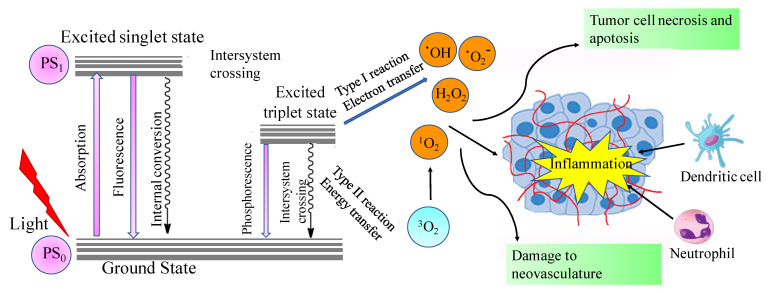
Modified Jablonski scheme illustrating the principles and mechanisms of photodynamic therapy. Reprinted with permission from Ref. [8]. Copyright 2006 Springer Nature.

Photodynamic therapy (PDT) is a novel minimally invasive treatment option that has been used to treat a wide variety of cancers and other diseases [1,2,3,4]. PDT includes a photosensitizer that is inactive in the dark. When irradiated with visible light, usually long wavelength red light, in the presence of oxygen, the photosensitizer will be activated to act as an energy transducer, transferring energy to molecular oxygen (Figure 1). This transfer results in the generation of a series of highly reactive oxygen species (ROS), such as singlet oxygen (^1^O_2_) [5,6,7]. PDT destroys cancer tissues via three mechanisms: (1) During light irradiation, ROS species are generated, which will kill cancer cells directly; (2) PDT will destroy a tumor through irreversible damage to the tumor vasculature; and (3) PDT will stimulate the immune response directed against tumor cells [5,6,7,8] (Figure 1). Based on these mechanisms, PDT has several advantages for cancer treatment, including its minimal invasiveness, targeted toxicity of the defined tumor tissues using targeted visible light irradiation, and the ability to bypass the several resistance mechanisms displayed by malignant cells [9,10,11].

**Figure 2:**
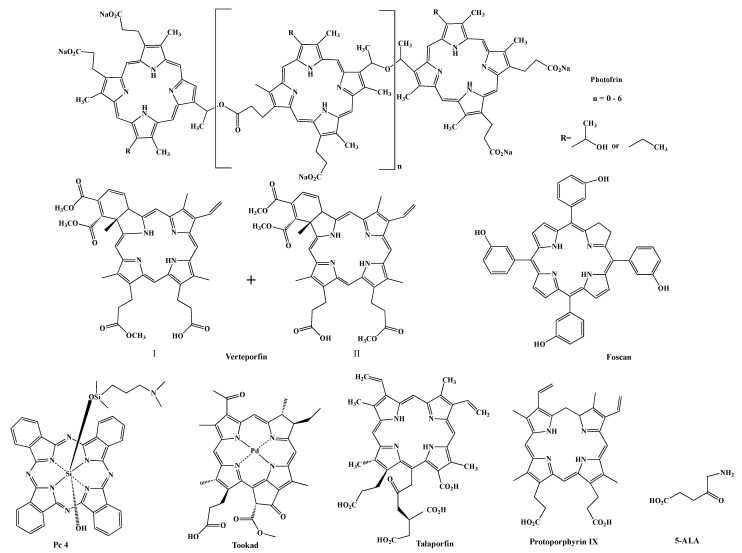
Structures of some photosensitizers and protoporphyrin IX precursor 5-ALA.

Various photosensitizers have been reported. Photofrin (porfimer sodium) (Figure 2) is the first approved PDT agent for the treatment of cancer and is still widely used to treat lung, esophageal, bladder cancers, and cervical cancer [12,13,14,15]. The limitations of Photofrin include its lack of purity, poor tissue penetration at the activation light (630 nm) and prolonged skin phototoxicity [16]. Theses drawbacks encouraged the development of second-generation PDT agents, including macrocyclic compounds, such as Verteporfin [17], which is a 1:1 mixture of regioisomers, Foscan (mTHPC) [18], silicon phthalocyanine 4 (Pc 4) [19], Tookad [20], Talaporfin and protoporphyrin IX (PPIX) precursors, such as 5-aminolevulinic acid (5-ALA, Levulan) [21] (Figure 2). Compared to Photofrin, most of the second-generation PSs are pure single compounds and have high singlet oxygen quantum yields (Table 1). Pc 4 also has a very high quantum yield for fluorescence, enabling it to also be utilized for theranostic approaches, see below. They are excited at longer wavelengths, which allows deeper penetration of the light into the tissue and can be used to treat deep-seated tumors, resulting in improved treatment efficacy. The main drawbacks associated with second-generation photosensitizers are poor water solubility and slow body clearance rate. Although 5-ALA shows high tumor selectivity to glioblastoma and bladder cancers, the lack of selectivity to desired targets remains a problem for most photosensitizers. While specific delivery of light to the tumors renders selectivity to PDT treatment, most photosensitizers are non-selectively distributed in the body and will cause side effects, e.g., accumulation in skin, causing patients to shield themselves from bright light for several days. The development of third-generation PSs aimed to improve the pharmacological characteristics and tumor selectivity. PSs have shown improved tumor accumulation when incorporated into delivery systems, such as liposomes [22,23,24,25], micelles [26,27,28], gold nanoparticles [29,30,31], and silica-based nanoparticles [32]. Approaches of using nanoparticles to passively deliver photosensitizers to tumors have been extensively reviewed by others [33,34,35] and will not be discussed here. The most promising results are obtained from studies that actively targeted antigens or biomolecules that are over expressed on cancer cells or tumor vasculature. In this review, we will discuss recent developments of targeting PDT treatment, either through direct attachment of a targeting moiety to PSs or through attachment of a targeting moiety to delivery systems.

## 2. Targeting via Antibody

**Figure 3:**
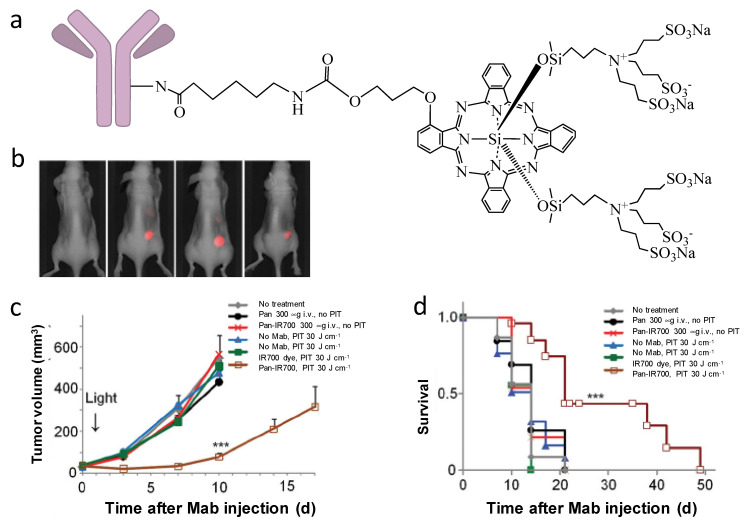
(**a**) General structure of antibody-based IR700 conjugates for NIR-PIT. (**b**) Pan-IR700 selectively accumulate in EGFR-expressing A431 tumors (right), but not in EGFR-non-expressing 3T3 tumors (left). (**c**) PIT with Pan-IR700 significantly inhibited A431 tumor growth. (**d**) PIT with Pan-IR700 significantly prolonged animal survival time. (***: *p* < 0.001). Reprinted with permission from Ref. [62]. Copyright 2011 Springer Nature.

The advances of monoclonal antibodies provide opportunities to use their specific binding properties for targeted drug delivery. The approach using antibodies to specifically conjugate to PSs for targeted PDT is called photoimmunotherapy (PIT). Among the PSs for PIT, phthalocyanine dye IR700 is the most widely used. Compared to other photosensitizers, IR700 has excellent water solubility and it emits light at 700 nm when irradiated by 690 nm light; therefore, IR700 can serve both as a diagnostic tool (fluorescence imaging) and as a therapeutic approach (PDT). In 2011, Mitsunaga et al. first reported near-infrared photoimmnuotherapy (NIR-PIT) by conjugating IR700 to monoclonal antibodies (mAb) [62]. Conjugation of IR700 to epidermal growth factor receptors (EGFR)—targeting panitumumab (Pan-IR700) showed selective uptake in EGFR expressing A431 cells and effective tumor shrinkage after irradiation was observed in EGFR expressing A431 tumors (Figure 3). Mab-IR700 was most effective when bound to the cell membrane, while non-bound mab-IR700 had no phototoxicity. It has been found that NIR-PIT induces immunogenic cell death (ICD), which elicits a host immune response against tumor [63,64] potentially adding to its efficacy to eradicate cancer. Interestingly, ICD-induced morphological changes are only observed in target-expressing cells, but not in target-non-expressing cells, which is different from traditional PDTs that cause apoptosis/necrosis in both targeted cells and adjacent non-targeted cells and normal tissues [63,65,66]. 

Presently, various antibodies have been tested for NIR-PIT, including anti-CEA-IR700 for pancreatic cancer [67], anti-CD47 for bladder cancer [68], anti-PSMA for prostate cancer [69,70], anti-CD44 for oral cancer [71], anti-DLL3 for small cell lung cancer [72], and anti-CD133 for glioma [73] (Table 2). All of these IR700-based mAb conjugates showed the ability to selectively bind to and kill cancer cells that express the target antigen. No phototoxicity was observed in adjacent non-expressing cells. Excitingly, an EGFR-targeted antibody cetuximab-IR700 conjugate RM-1929 has entered global phase III trials for the treatment of head and neck cancers [60,61]. 

**Figure 4:**
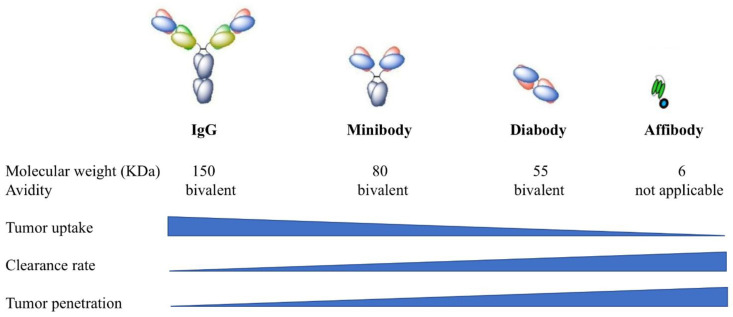
Comparison of full-length antibodies (IgG), minibodies, diabodies, and affibodies. Reprinted with permission from Ref. [85]. Copyright 2018 Chemistry Europe.

While great success has been achieved with full-length antibodies, antibodies do not penetrate evenly into tumor parenchyma, due to their relatively large size which limits the effectiveness of therapy [74,75]. Recently, smaller antibody fragments, such as diabodies and minibodies have been developed as alternatives for full-length antibodies [76]. Diabodies are bivalent scFV dimers [77] and minibodies are bivalent dimers of scFv-CH_3_ fusion proteins [78]. In contrast to full antibodies (~150 kDa), diabodies (~55 kDa) and minibodies (~80 kDa) are much smaller, but retain the essential specificities and affinities of full antibodies (Figure 4). Watanabe et al. compared the effectiveness of PIT using diabody (Db-IR700) and minibody (Mb-IR700) against prostate specific membrane antigen (PSMA) to PIT using full length IgG antibody against PSMA (IgG-IR700) [79]. In in vitro studies, selective uptake of IR700 was observed in the cell membrane of PSMA-positive cells when incubated with IgG-IR700, Db-IR700 or Mb-IR700, and rapid phototoxic cell death was observed after NIR light irradiation. In contrast, no localization of IR700 was observed when PSMA-negative cells were incubated with the conjugates, and the cells were not killed by exposure to NIR light. Biodistribution studies using ^125^I-labeled IgG, Db and Mb showed that the peak accumulation time in PSMA-positive tumors was 24 h for IgG-IR700 and Mb-IR700 and 6 h for Db-IR700. While different pharmacokinetic profiles were observed between the three conjugates, equal effectiveness of photoimmunotherapy was observed with both full-length IgG-IR700 and antibody fragment Db-IR700 and Mb-IR700. Therefore, the use of Db-IR700 shortened the time interval between injection and NIR exposure, which potentially will aid in clinical application. The other alternative for a full-length antibody is an affibody. Affibody molecules are engineered proteins that have a 58-amino acid sequence folded into three alpha helices [80,81]. Affibody molecules have very small size with a molecular weight at about 6–7 kDa, therefore, they have a much shorter circulation time. Burley et al. conjugated an EGFR specific affibody Z_EGFR:03115_ to IR700 and tested it in a glioblastoma model [82]. Z_EGFR:03115_-IR700 showed significant activity in inducing cell death in EGFR-positive U87-MGvIII glioblastoma cells in vitro. Consistent with previous mAb-IR700 treatment, necrotic cell death was observed within 1 h post PIT treatment. In vivo imaging studies demonstrated clear tumor visualization as early as 1 h post injection in both subcutaneous and orthotopic U87-MGvIII tumor models, while that for intact antibodies took at least 24 h. Again, in vivo PIT using Z_EGFR:03115_-IR700 significantly inhibited U87-MGvIII tumor growth. Furthermore, similar to mAb-IR700, affibody-IR700 conjugates can also induce ICD after light irradiation [83]. These studies demonstrated that replacement of full-length antibodies with smaller fragment of antibodies can achieve effective PIT with improved pharmacokinetics [79,82,83,84].

## 3. Targeting by Small Ligands and Peptides

Compared to antibodies, small organic ligands and peptides have excellent tumor penetration properties, which in combination with their selective binding and rapid internalization, make them ideal alternatives to antibodies for tumor targeting applications. Unlike antibodies, the small ligands/peptides themselves are less immunogenic than the larger antibodies and are expected to have minimal side effects [86]. The most promising compounds include folic acid derivatives targeting the folate receptor (FR), glutamic acid urea compounds targeting PSMA, cyclic peptides against integrin α_v_β_3_, somatostatin analogues targeting somatostatin receptor (SSTR), and aromatic sulfonamides specific to carbonic anhydrase IX (CAIX). For targeted delivery of PDT, these ligands can be either conjugated directly to photosensitizers, or can be conjugated to the delivery systems for photosensitizers.

### 3.1. Folate Receptor

Folate receptors (FR) are folate-binding membrane proteins that are overexpressed in many solid tumors [87,88]. FRs bind folate with high affinity. Folic acid derivatives (FA) represent the first small molecule ligands that have been successfully used for selective delivery of chemotherapeutic and imaging agents to cancer cells [89]. Targeted PDT using folic acid derivatives also appears to be a promising treatment for various cancers [90,91,92,93,94,95]. Wang et al. conjugated FA with pyropheophorbide (Pyro) [92]. Compared to free Pyro, increased cellular uptake of Pyro was observed when cells were incubated with FA-Pyro. FA-Pryo also improved the treatment efficacy of PDT. With only one or two light irradiations, KB tumors were eradicated. The FA-Pyro conjugate was also reported to be effective on ovarian cancer [95]. It was found that PDT with FA-Pyro can activate the immune response by inducing the secretion of immunoactivating cytokines (IL2 and IFNγ), reducing the production of immunosuppressive cytokines (TGFβ) and releasing extracellular vesicles which are prone to activating immune cells. The authors also showed that FA-Pyro PDT at the tumor can activate CD4+ and CD8+ T cells, indicating that FA-Pyro-based PDT can elicit antitumor immune response.

**Figure 5:**
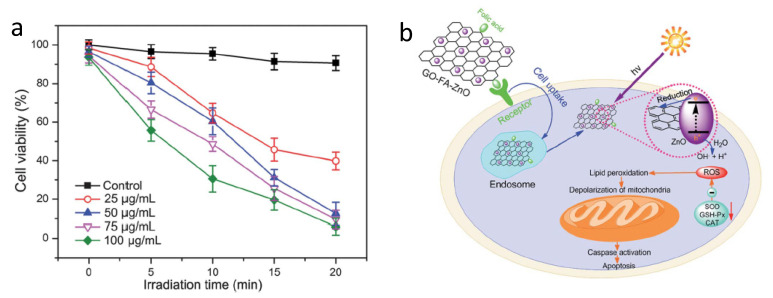
(**a**) Phototoxicity of GO–FA–ZnO. (**b**) Hypothetical mechanism of GO–FA–ZnO-induced PDT. Reprinted with permission from Ref. [96]. Copyright 2013 Royal Society of Chemistry.

In addition to direct conjugation to PSs, folic acid has also been used to target delivery systems for targeted delivery of PSs. Huang et al. designed and prepared a novel water-soluble folic acid-graphene oxide (FA-GO) system for targeted PDT [91]. The FA-GO existed in sheet-like shapes with a thickness of about 1.2 nm. The PS Chlorin e6 (Ce6) was then loaded into the system. The large surface area of GO resulted in a loading efficacy as high as 80%. Compared to non-targeted GO-Ce6, FA-GO-Ce6 significantly increased selective accumulation of Ce6 in MGC803 stomach cancer cells and light irradiation caused 90% cell death, indicating the potency of PDT. Similar results were reported with folic acid modified graphene oxide hybrid loaded with zinc oxide (FA-GO-ZnO) [96]. PDT by FA–GO-ZnO (Figure 5) generated reactive oxygen species (ROS), which significantly reduced cell viability. Moreover, increased caspase 3 activity was observed after PDT, indicating the apoptotic cell death induced by PDT.

Porphysomes are recently developed liposome-mimicking nanoparticles that self-assemble from porphyrin–phospholipid conjugates [97]. They have extremely high porphyrin density (>80,000 per nanoparticle). The porphysome bilayers can increase the efficacy of PS delivery and improve PDT efficacy. Due to the dense packing, the photoactivity (fluorescence and generation of singlet oxygen) of porphyrin is quenched. Porphysomes have been modified by folic acid (folate–porphysome, FP) to enable targeting to the folate receptor [94,98]. By FA-mediated endocytosis, FP was internalized into FR-positive cells rapidly resulting in a 58.7-fold enhanced uptake compared to non-targeting porphysomes. Fluorescence signal from porphyrin was observed after the FR-positive cells was incubated with FP for 24 h, in contrast, no fluorescence was observed when the FR-positive cells were incubated with non-targeting porphysomes, indicating that FR-targeting results in disassembly of FP nanostructure and subsequence fluorescence activation. Further, in vitro and in vivo studies showed FR-selective PDT efficacy. Therefore, FR-targeting triggered nanostructure disruption providing an activation method to de-quench the tightly packed porphyrin, enhancing PDT efficacy.

### 3.2. Prostate Specific Membrane Antigen

Prostate specific membrane antigen (PSMA) is a type-II transmembrane protein that is highly overexpressed in prostate cancers [99,100,101,102,103,104]. PSMA’s transmembrane location and internalization make it an ideal target for imaging and therapy [105,106,107,108,109,110]. The first PSMA-targeting PDT was reported in 2009 by Liu et al. [111] who used a peptidomimetic inhibitor of PSMA to conjugate the porphyrinic photosensitizer, pyropheophorbide-a. Among the PSMA ligands, the glutamic acid urea derivative gained most interest due to their high affinity for PSMA, specificity for PSMA, and fast and efficient internalization in PSMA-positive cells [112,113,114,115,116,117]. We have developed a unique highly negatively charged PSMA ligand based on the fact that arginine-rich S1 binding pocket is highly positively charged [118,119]. The ligand, PSMA-1, is rationally designed to include three D-glutamic acids in the structure. The D-glutamic acids will form strong ion pairs with the positively charged guanidine groups of arginine in the substrate binding pocket of PSMA to improve the binding affinity [106]. We have conjugated it to a Pc 4 derivative (Pc413) and IR700 [105]. These two conjugates demonstrated selective and specific uptake in PSMA-positive PC3pip cells. Both can effectively inhibit PC3pip tumor growth after NIR light irradiation. Others have tried to conjugate PSMA ligand to pyropheophorbide [120] and bacteriochlorophyll [121]. A nine D-peptide-linker was inserted between the PSMA ligand and the PSs to prolong the plasma circulation time of the conjugates (12.65 h). Over the 24-h post conjugate administration, an increased fluorescence ratio between PSMA-positive PC3pip and PSMA-negative PC3flu tumors was observed, which reached 3.1 at 24 h. The improved tumor accumulation led to an effective PDT in PC3pip tumors.

**Figure 6:**
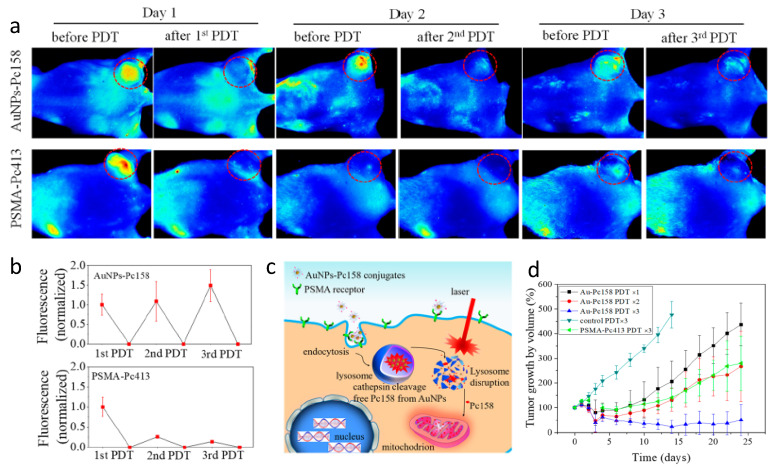
(**a**) Comparison of fluorescence images of mice received PSMA-targeting AuNPs-Pc158 and PSMA-1-Pc413 before and after each PDT. (**b**) Normalized fluorescence intensity of PSMA-targeting AuNPs-Pc158 and PSMA-1-Pc413. (**c**) Illustration of lysosome release of Pc158 by cathepsin and accumulation of Pc158 into mitochondria. (**d**) Multiple PDT with PSMA-targeting AuNPs-Pc158 effectively inhibits large tumor (>500 mm^3^) growth. Reprinted with permission from Ref. [123]. Copyright 2020 American Chemical Society.

In addition to simple molecule PSMA-1-Pc413, Mangadlao et al. synthesized PSMA-targeting gold nanoparticles (AuNP) for targeted delivery of Pc 4, in which Pc 4 was non-covalently absorbed into pegylated AuNPs [122]. Although PSMA-targeted AuNP-PEG5K-PSMA-Pc4 accumulated four times more in PC3pip tumor than in PC3flu tumor as measured by gold nanoparticle accumulation, the difference of the fluorescent from Pc4 between PC3pip and PC3flu was not that significant. More recently, Luo et al. improved the nanoparticle system by covalently conjugating a Pc 4 derivative, Pc158, to AuNP through a cathepsin cleavable linker [123]. These studies showed that covalent conjugation of Pc158 to AuNPs improved the selectivity and fluorescence discrimination between PSMA positive and negative tumors. One interesting phenomena of the PSMA-targeted AuNP-Pc158 was the recovery of fluorescence in the tumor after PDT (Figure 6). Following irradiation, which bleaches out fluorescence from the PDT agent, fluorescence of the tumors recovered and even increased without more administration of the PSMA-targeted AuNP-Pc158. In contrast, the small molecule PSMA-1-Pc413 showed good accumulation in the tumor at 24 h, but no Pc413 fluorescence recovery was observed after one light PDT irradiation. As a result, sequential PDT after a single administration of PSMA-targeted Au-NPs was more potent in inhibiting PC3pip tumor growth as compared to mice that received PSMA-1-Pc413 with sequential PDTs. Careful quantification of targeted tumor fluorescence and gold content indicated that there was a time-dependent release of Pc158 from the nanoparticles into the tumor cells and a time dependent PDT-induced increase of the targeted nanoparticles into the tumors due to their long blood half-life. Delivery of Pc158 via PSMA-targeted gold nanoparticles improved tumor accumulation of Pc158 via several mechanisms, resulting in significant tumor growth inhibition as compared to small molecule delivery, i.e., PSMA-1-Pc413.

As a final note, PSMA is overexpressed in the neovasculature of most solid human tumors and potentially provides a biomarker for PSMA-targeted PDT for a number of human tumors [124,125].

### 3.3. Integrin α_v_β_3_

**Figure 7:**
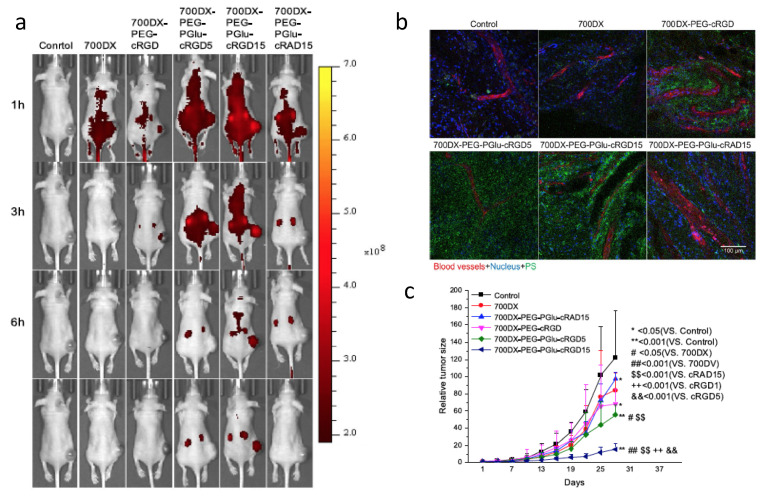
(**a**) Fluorescence images of mice bearing U87MG tumors after injected with arginine-glycine-aspartate peptide (RGD) modified IR700-polymers. (**b**) Confocal images showing intratumor distribution of photosensitizers. (**c**) In vivo PDT of mice bearing U87MG. Reprinted with permission from Ref. [137]. Copyright 2018 Springer Nature.

Tumor angiogenesis supplies oxygen and nutrients to tumors. It has an important role in tumor progression as well as the development of metastasis [126]. Anti-angiogenesis has become an effective therapy for cancer treatment. Integrin α_v_β_3_ is a heterodimer that plays an important role during tumor angiogenesis [127]. The protein is over expressed not only on activated tumor endothelial cells but also on tumor cells, allowing for anti-integrin therapy targeting both tumor vasculature and tumor cells [128,129]. Among the integrin inhibitors, the cyclic arginine-glycine-aspartate peptide (cRGD) is the best known [130,131,132,133]. Photosensitizers protoporphyrin IX [134] and pyropheophorbide [111,135] have been successfully conjugated to cRGD, which demonstrated successful targeting and improved PDT efficacy. Li et al. conjugated cRGD and IR700 to albumin [136]. When TOV21G ovarian cancer cells were incubated with the cRGD modified albumin nanoconjugates, cellular delivery of IR700 was increased 121-fold as compared to cells incubated with control nanoconjugates. Dynamin-mediated caveolae-dependent endocytosis pathways were suggested to be involved in integrin-targeted IR700 delivery. Phototoxicity was also found to be specific to integrin. These studies indicated that modifying the surface of nanoparticles by targeting ligands can selectively deliver the nanoparticles to receptors. Dou et al. studied the numbers of cRGD peptides on IR700 conjugated polymers (Figure 7), and found that the accumulation of IR700 in the tumor increased with increased number of cRGDs on the polymers [137]. Monomeric cRGD (700DX-PEG-PGlu-cRGD) showed some improvement to increase the accumulation of 700DX; however, the accumulation of IR700 was much less compared with 700DX-PEG-PGlu-cRGD5 and 700DX-PEG-PGlu-cRGD15. Microscopic studies of tumors found accumulation of RGD targeting polymers not only within the cells but also on tumor-associated vasculature. The accumulation of IR700 on vasculature increased when more cRGDs was incorporated on the polymers. These results suggest that the number of cRGD peptides can control intratumoral distribution pattern of photosensitizers.

### 3.4. Somatostatin Receptor

Somatostatin receptors (SSTRs) are transmembrane proteins that belong to the G-protein coupled receptor (GPCRs) family and are responsible for translating extracellular signals to intracellular responses [138]. SSTRs, especially SSTR subtype 2 (SSTR2) are found expressed at relatively higher levels in many tumor cells and in tumor blood vessels relative to normal tissues [139]. Upregulated SSTR in tumors makes it an attractive cellular target for PDT, since a photosensitizer-conjugate can be used to target tumor cells as well as neovasculature. Somatostatin and its analogues bind to SSTR with high binding affinity in the nanomolar range [139]. Octreotate and octreotide are cyclic peptides containing two D-amino acids. Compared to natural somatostatin, they have improved serum stability [140]. Starkey et al. conjugated octreotate to a porphyrin-based photosensitizer to target tumor vasculature [141]. It was found that SSTR-targeted PDT led to tumor vascular shutdown, while untargeted PDT or EGFR-targeted PDT failed to produce an adequate vascular response. Conjugation of a cyclometalated luminescent Ir(III) complex to octreotide showed selective and specific uptake and improved phototoxicity in SSTR-positive cells Hela cells [142]. Similar results were observed with SSTR targeting Ce6-octreotate conjugate [143] and somatostatin–ruthenium (II) polypyridine conjugate [144,145].

### 3.5. Carbonic Anhydrase IX

As stated before, oxygen is one of the essential components for PDT. During light activation, the excited photosensitizers (PSs) will transfer energy to molecular oxygen and generate singlet oxygen (^1^O_2_) and other ROS species, which can damage biomolecules causing them to initiate cell death [5,6,7]. Therefore, sufficient oxygen is needed for successful PDT. Unfortunately, tissue hypoxia is a key feature of many solid tumors [146]. Fast consumption of PDT further aggravates the hypoxic condition, reducing the efficacy of PDT [147]. Carbonic anhydrase IX (CAIX) is constitutively up-regulated in solid tumors and its overexpression in cancer tissues is strongly regulated by hypoxia [148]. Attempts have been tried to overcome the effect of hypoxia by targeting CAIX using small aromatic sulfonamide inhibitor of CAIX [149,150]. For example, an acetazolamide (AZ)-conjugated BODIPY photosensitizer (AZ-BPS) was designed and synthesized [150]. AZ-BPS showed improved uptake and greater phototoxicity in CAIX-positive MDA-MB-231 cells than CAIX-negative MCF-7 cells. In addition, AZ-BPS was more than 142-fold more potent than untargeted BPS against MDA-MB-231 cells. It was also found that AZ-BPS induced cell death through mitochondria dysfunction. In vivo studies showed that PDT by AZ-BPS significantly inhibited tumor growth compared to PDT by BPS. PDT by AZ-BPS decreased the levels of angiopoietin-2 (ANGPT2) and vascular endothelial growth factor A (VEGFA) which promote the initiation of angiogenesis and maturation of new vessels. In contrast, PDT by BPS increased the VEGFA expression, which indicated the resistance to BPS-induced PDT. Therefore, PDT targeting CAIX provided both anti-angiogenesis and PDT effects to the hypoxia tumors, achieving a better treatment outcome.

## 4. Conclusions and Perspectives

Photodynamic therapy has become an effective alternative to traditional anticancer therapy. PDT has shown efficacy in patients with inoperable cancers and have extended patients’ overall survival time in clinical studies. Compared to chemotherapy and radiotherapy, PDT-based cancer treatment significantly reduces side effects and improves target specificity because only the lesion under light irradiation is treated. The selectivity of PDT can be further improved by targeted delivery of photosensitizers to cancer cells to improve their selectivity to tumor tissues and to reduce their accumulation in normal tissues. In this review, we briefly reviewed recent developments of targeted PDTs including targeting through antibody and targeting using small molecular ligands or peptides. Clearly, the development of novel photosensitizers with tumor-specific properties leads to more effective PDT and new applications for these drugs. Currently, cetuximab-IR700 conjugate RM-1929 has entered phase III clinical trials.

In addition to treatment, diagnosis and prognosis are also important in winning the war against cancer. The fluorescence properties of photosensitizers can be exploited for fluorescence diagnostic imaging; this technology is also known as photodynamic diagnosis (PDD). PDD and PDT enable the diagnosis and simultaneous treatment of the cancer and permit a real-time follow-up of the progress of the disease, which is also known as “theranostics” [151]. Fluorescence image-guided surgery (FIGS) of glioblastoma using non-targeted photofrin and 5-ALA was first reported in 2000 [152]. Later on, a new strategy combing FIGS and PDT was reported [153,154]. However, in both cases, non-targeted photofrin and 5-ALA were used. Molecular targeting plays an important role in theranostics [151], it is imperative to develop more specific photosensitizers which can specifically and differentially diagnose cancer cells and kill them. Since the quantum yield of light from Pc 4, and presumably its Pc413 derivative, was so high (Table 1), Wang et al. demonstrated that PSMA-targeted Pc413 could be utilized for fluorescence image-guided surgery (IGS), adding to the utility of PSMA-targeted PDT [155]. IGS achieved more complete tumor resection compared to white light surgery (WLS). More importantly, PDT after IGS showed significantly delayed tumor recurrence and extended animal survival as compared to WLS and IGS groups. Therefore, PSMA-1-Pc413 can be used as an effective adjuvant therapy after image-guided surgery to destroy unresectable cancer tissue or missing cancer cells, reducing the frequency of positive margins and tumor recurrence. Finally, in orthotopic prostate cancer models it was also shown that the PSMA-targeted Pc413 could identify prostate cancer cells within the lymph nodes, providing novel avenues for further study.

Due to the unique mechanisms of PDT, it has been utilized in combination with chemotherapy to overcome chemo-resistant cancers and achieve a synergistic therapeutic effect with chemotherapy [9,156,157,158,159,160]. Although intriguing results have been found for the treatment of cancer by combination of PDT and chemotherapy, both are limited by off-target tissue accumulation leading to cell death in normal tissue. Ito et al. reported a T−DM1−IR700 conjugate, in which both photosensitizer IR700 and chemotherapeutic drug DM-1 were conjugated to the antibody Trastuzumab [161]. The conjugate therefore can achieve both NIR-PIT and chemotherapy. In small tumor models, T-DM1-IR700 did not show improved antitumor activity when compared to T-IR700. However, T-DM1-IR700 was more effective in large tumor models which could not receive sufficient NIR light. More recently, a multifunctional mesoporous CuS nanoplatform (FA-CuS/DTX@PEI-PpIXCpG nanocomposites) targeting folate receptor was reported [162]. The smart nanoplatform combined PDT, photothermal therapy (PTT) and docetaxel (DTX)-enhanced immunotherapy. A synergistic effect was observed, which resulted in highly superior antitumor activity in triple negative 4T1 tumors. These studies indicated that targeted PDT in combination with other treatments can be a new treatment options for caner.

The other important perspective of PDT is antitumor immune responses induced by PDT [5,6,7]. PDT-induced immune response first occurs in the treated area, then extends throughout the body. Therefore, PDT is not only a simple local therapy, but it can also have systemic effects. PIT has been reported to be effective against metastatic diseases with PDT only administered to the main tumor mass [60,163,164].

Finally, it has been reported that cells can develop resistance during PDT [165,166,167]. Mechanisms of resistance to PDT include upregulation of antioxidant and anti-apoptotic proteins [168], enhanced activity of membrane transporter ATP-binding cassette G member 2 (ABCG2) to efflux 5-ALA-induced PPIX and other photosensitizers from cells [169]. Increased levels of endogenous nitric oxide have also been reported to induced cell resistance to PDT with 5-ALA [170,171]. It needs to be noted that although targeted PDT can enhance the efficacy of PDT, cancer cells may still develop resistance to the treatment. Different strategies will be needed to overcome resistance to PDT.

In summary, great achievements have been made with targeted PDTs. They represent suitable therapeutic alternatives and have great potential for the treatment of a wide variety of cancers. More targeted photosensitizers are expected to move to clinical trials in the future due to their therapeutic enhancements including efficacy, specificity, marginal toxicity to normal cells, and minimal side effects. 

## Figures and Tables

**Table 1:** Summary of photosensitizers in clinical use.

Photosensitizer	Absorption (nm)	Extinction Coefficient (ε, M^−1^cm^−1^)	Fluorescence Quantum Yield (Φ_F_)	Single Oxygen Quantum Yield (Φ_∆_)	Cancer Types
Photofrin	630	3000 in methanol (MeOH)	0.05 in MeOH [36]	0.35 in MeOH [37]	Lung cancer, esophagus, bile duct cancer, bladder cancer, glioblastoma, ovarian cancer, breast cancer, skin metastases [38]
Verteporfin	690	34,000 in MeOH	0.051 in MeOH [39]	0.77 in MeOH [37]	Pancreatic cancer, breast cancer [17]
Foscan	652	40,000 in MeOH	0.18 in MeOH	0.42 in MeOH [40]	Squamous cell carcinoma, head and neck carcinoma [41,42]
Pc4	672	20,000 in acetonitrile	0.4 in acetonitrile	0.43 in acetonitrile [43,44]	Squamous cell carcinoma, basal cell carcinoma [45]
Tookad	753	100,000 in water [20]	NA ^a^	NA	Prostate cancer, Esophagus [20,46]
Talaporfin	664	40,000 in phosphate buffered saline (PBS)	0.19 in PBS	0.77 in PBS [47]	Early lung cancer, malignant brain tumor, refractory esophageal cancer, liver metastasis, colorectal neoplasms [48,49,50,51]
Levulan ^b^	630	3000 in MeOH	0.06 in MeOH [52]	0.56 in 1–3% TX100 [53]	Skin cancer, bladder cancer, glioblastoma, Esophagus [21,54,55]
Metvix ^b^	630	3000 in MeOH	0.06 in MeOH [52]	0.56 in 1–3% TX100 [53]	Skin cancer [56]
Hexvix ^b^	630	3000 in MeOH	0.06 in MeOH [52]	0.56 in 1–3% TX100 [53]	Bladder cancer [57]
RM-1929 (cetuximab-IR700 conjugate)	689	210,000 in PBS	0.24 in PBS [58]	0.3 in PBS [59]	Head and neck cancer [60,61]

^a^: NA: not available; ^b^: data are based on 5-ALA-induced protoporphyrin IX.

**Table 2:** Summary of IR700-based photoimmunotherapy.

PIT Conjugate	Antibody	Targeting Antigen	Tumor Type	References
Tra-IR700	transtuzumab	Epidermal growth factor receptor 2 (EGFR 2) (HER2)	3T3/HER2 mouse embryonic fibroblasts	[62]
Pan-IR700	panitumumab	Epidermal growth factor receptor 1 (EGFR 1) (HER1)	A431 epidermal carcinoma	[62]
Anti-CEA-IR700	Anti-CEA mAb	Carcinoembryonic antigen (CEA)	BxPC3 pancreatic cancer	[67]
Anti-CD47-IR700	Anti-CD47 mAb B6H12	CD47	639V bladder cancer	[68]
Anti-PSMA-IR700	Anti-PSMA mAb	Prostate specific membrane antigen (PSMA)	PC3pip prostate cancer	[69]
Anti-CD44-IR700	Anti-CD44 mAb IM7	CD44	MOC1 and MOC2 oral cavity squamous cell carcinoma	[71]
Rova-IR700	rovalpituzumab	Delta-like protein 3 (DLL3)	SBC5 small cell lung cancer	[72]
AC133-IR700	AC133 mAb	CD133	CD133-OE U251 glioma tumor and NCH421k glioblastoma stem cell	[73]
RM-1929	Cetuximab	Epidermal growth factor receptor (EGFR)	Head and neck cancer	[60,61]
